# Biophysical model of muscle spindle encoding

**DOI:** 10.1113/EP091099

**Published:** 2023-03-26

**Authors:** Stephen N. Housley, Randal K. Powers, Paul Nardelli, Sebinne Lee, Kyle Blum, Guy S. Bewick, Robert W. Banks, Timothy C. Cope

**Affiliations:** ^1^ School of Biological Sciences Georgia Institute of Technology Atlanta GA; ^2^ Department of Physiology and Biophysics University of Washington Seattle WA USA; ^3^ Department of Physiology, Feinberg School of Medicine Northwestern University Chicago IL USA; ^4^ Institute of Medical Science University of Aberdeen Aberdeen UK; ^5^ Department of Biosciences Durham University Durham UK; ^6^ W. H. Coulter Department of Biomedical Engineering Emory University and Georgia Institute of Technology, Georgia Institute of Technology Atlanta GA

**Keywords:** biophysical modelling, muscle spindle firing, sensory encoding, voltage‐gated ion channels

## Abstract

Muscle spindles encode mechanosensory information by mechanisms that remain only partially understood. Their complexity is expressed in mounting evidence of various molecular mechanisms that play essential roles in muscle mechanics, mechanotransduction and intrinsic modulation of muscle spindle firing behaviour. Biophysical modelling provides a tractable approach to achieve more comprehensive mechanistic understanding of such complex systems that would be difficult/impossible by more traditional, reductionist means. Our objective here was to construct the first integrative biophysical model of muscle spindle firing. We leveraged current knowledge of muscle spindle neuroanatomy and in vivo electrophysiology to develop and validate a biophysical model that reproduces key in vivo muscle spindle encoding characteristics. Crucially, to our knowledge, this is the first computational model of mammalian muscle spindle that integrates the asymmetric distribution of known voltage‐gated ion channels (VGCs) with neuronal architecture to generate realistic firing profiles, both of which seem likely to be of great biophysical importance. Results predict that particular features of neuronal architecture regulate specific characteristics of Ia encoding. Computational simulations also predict that the asymmetric distribution and ratios of VGCs is a complementary and, in some instances, orthogonal means to regulate Ia encoding. These results generate testable hypotheses and highlight the integral role of peripheral neuronal structure and ion channel composition and distribution in somatosensory signalling.

## Abstract (highlights)

- **What is the central question of the study?**
  How does the neuronal architecture and asymmetric distribution of voltage‐gated channels influence mechanosensory encoding by muscle spindle afferents?
- **What is the main finding and its importance?**
  The results predict that neuronal architecture and the distribution and ratios of voltage‐gated ion channels are a complementary and, in some instances, orthogonal means to regulate Ia encoding. The importance of these findings highlights the integral role of peripheral neuronal structure and ion channel expression in mechanosensory signalling. Generally, our computational approach offers an integrative means to generate testable hypotheses and prioritize targets for future mechanistic studies.

## 1 INTRODUCTION

Muscle spindles encode mechanosensory information by mechanisms that remain only partially understood. Long‐running study exposes complex multiformity and interdependence among molecular mechanisms that challenge a simplistic comprehensive explanation of how muscle spindle afferents generate the spike‐train patterns that encode mechanical responses of skeletal muscles. Muscle spindle complexity expresses itself in mounting evidence of various molecular mechanisms that play essential roles in muscle mechanics, mechanotransduction and intrinsic modulation of muscle spindle firing behaviour (Bewick & Banks, 2015; Bewick et al., 2005; Blum et al., 2020; de Nooij et al., 2015; Kröger & Watkins, 2021; Simon et al., 2010; Woo et al., 2015). Voltage‐gated ion channels (VGCs), the focus of the present study, also express diversity in type and distribution across specialized neuronal architecture (Bewick & Banks, 2015; Bewick & Banks, 2021; Blecher et al., 2018; Carrasco et al., 2017). Non‐linearities, time dependencies, and interactions in mechanotransduction, encoding, and their modulation collectively defy intuitive reasoning as an effective approach to understanding either the net formulation of muscle spindle firing or the exact role of individual components. Biophysical modelling provides a tractable approach to achieve a more complete and realistic mechanistic understanding.

Assorted computational models developed within the past 50 years exhibit varying degrees of success in simulating firing behaviour of mammalian muscle spindles. Most models build upon mathematical descriptions of skeletal muscle mechanical properties and their first‐ or second‐order translation into receptor potentials followed by spindle afferent firing (Hasan, 1983; Lin & Crago, 2002; Mileusnic et al., 2006; Schaafsma et al., 1991). Some models promote mechanistic explanations by representing biophysical properties of muscle‐spindle subcomponents, including intrafusal muscle fibres and their distinctive mechanical properties as well as spike encoding sites and their occlusive interactions (Blum et al., 2020). By integrating simulated intrafusal muscle fibre cross‐bridge cycling, our recently developed spindle model advances biophysical explanation of dynamic spindle firing behaviour, specifically the initial burst (Blum et al., 2020). Largely missing in these models are details about potential neuronal factors regulating spindle firing such as the asymmetrically distributed VGCs and detailed neuronal architecture. Although some muscle spindle models introduce spike production by simple integrate and fire models (Niu et al., 2014), these approaches exclude the broader complement of VGCs uniquely distributed among the specialized neuroelectrical compartments. So far unexplained are the roles of newly identified subtypes of Na VGCs, for example, Na_v_1.6, 1.7, some intriguingly present in muscle spindle Ia terminals outside the region held responsible for action potential (AP) initiation and encoding (Carrasco et al., 2017). We expect that the asymmetric composition and spatial distribution of these and as yet undiscovered but anticipated VGCs impact spindle afferent firing, as they do in neurons throughout the peripheral, central and autonomic nervous systems (Lai & Jan, 2006).

In the present study, we aimed to construct a computational model of muscle spindle firing built on current knowledge of the neuronal architecture and asymmetric distribution of VGCs in mammalian muscle spindles. The model incorporates molecular‐level biophysical properties underlying two of the main processes determining muscle spindle firing. Model input derives from an advanced version of our recent muscle cross‐bridge model that computes muscle spindle mechanical responses to a variety of muscle stretch perturbations (Blum et al., 2020). Model output in the form of spike trains results from known conductance properties of multiple VGCs as they react to receptor potentials artificially represented by the computed mechanical responses. The model was designed to accommodate anticipated discovery of new VGCs as well as other key mechanisms in spindle input/output operations excluded from the present report, including mechanotransduction and feedback regulation of spindle firing, either intrinsic or extrinsic. The main objective of the present study was to examine the role of neuronal architecture and asymmetric distribution of VGC diversity by simulating the firing patterns extracted from our in vivo electrophysiological recording from adult rats (Housley et al., 2020, 2021; Vincent et al., 2017).

## 2 METHODS

### 2.1 in vivo electrophysiology and encoding parameters

All in vivo procedures have been previously described (Bullinger et al., 2011; Haftel et al., 2004; Housley et al., 2020; Nardelli et al., 2016, 2017; Vincent et al., 2017). Briefly, adult rats were deeply anaesthetized initially by inhalation of isoflurane (5% in 100% O_2_), and for the remainder of the experiment via a tracheal cannula (1.5−2.5% in 100% O_2_). Vital signs were continuously monitored including, core temperature (36–38°C), $P_{CO_{2}}$ (3–5%), respiratory rate (40−60 breaths/min), pulse rate (300–450 bpm) and $S_{pO_{2}}$ (>90%). Lumbar dorsal roots together with muscles and nerves in the left hindlimb were surgically exposed and prepared for stimulation and recording as previously described (Housley et al., 2020, 2022; Vincent et al., 2017). Individual axons in dorsal rootlets were penetrated by glass micropipettes and were selected for continuous intracellular study when electrical stimulation of triceps surae nerves produced orthodromic APs. Muscle spindle type Ia afferents were physiologically identified based on the following three criteria: firing that paused during the rising phase of isometric twitch force, fired with one‐to‐one fidelity during each cycle of muscle vibration (100 Hz frequency, 80 μm amplitude), and responded with an initial burst of high‐frequency firing (>100 pulses per second (pps)) at the onset of muscle stretch (20 mm/s). Spike trains generated by Ia neurons were measured for several primary and derived parameters reported in Results. Simultaneously recorded muscle kinetics were used as model inputs for our ‘Biophysical model of intrafusal fibre dynamics’ (see below).

### 2.2 Computational muscle spindle model structure

Three principal components of muscle spindle encoding were modelled: intrafusal fibre dynamics, receptor potential and spike generation. We simulated two parallel intrafusal muscle fibres – a fast chain fibre and a slower bag fibre. The receptor potential was then computed as a weighted sum of the force from the intrafusal fibre and the first time derivative of force. Spike generation was simulated with a four‐compartment biophysical neuron model whose physical dimensions and distribution of VGCs approximate the adult rat muscle spindles as estimated from confocal reconstructions and previously published studies (Carrasco et al., 2017).

#### 2.2.1 Biophysical model of intrafusal fibre dynamics

Following the structure of Blum et al. (2020) we modelled intrafusal fibres as a single half‐sarcomere whose force output is governed by cross‐bridge dynamics. Briefly, we used a validated, two‐state muscle model (MATMyoSim) to simulate the intrafusal fibres. The model includes two myosin states representing myosin attachment and detachment, as well as two states representing actin dynamics and actin–myosin cooperativity (Campbell, 2014), derived from the original Huxley muscle model equations (Huxley, 1957).

#### 2.2.2 Receptor potential generation

We used a phenomenological model of the mechanotransduction process, whereby receptor potentials were generated by directly converting the forces from the bag and chain fibres detailed previously (Blum et al., 2020). Briefly, the bag component of the receptor potential was computed as the weighted sum of the half‐wave rectified force and yank (first time derivative of force) from the bag fibre. The chain component of the receptor potential was computed as a weighted half‐wave rectified force from the chain fibre. Model parameter weights on the force from the bag ($k_{f_{b}}$), and yank from the bag ($k_{y_{b}}$) and from the chain force ($k_{f_{c}}$) were held fixed for all simulations. Components are then scaled by 2e^5^ to account for the transformation from force signals to receptor potential signals and summed together to get the final receptor potential.

#### 2.2.3 Biophysical model of neuron spike generation

**FIGURE 1:**
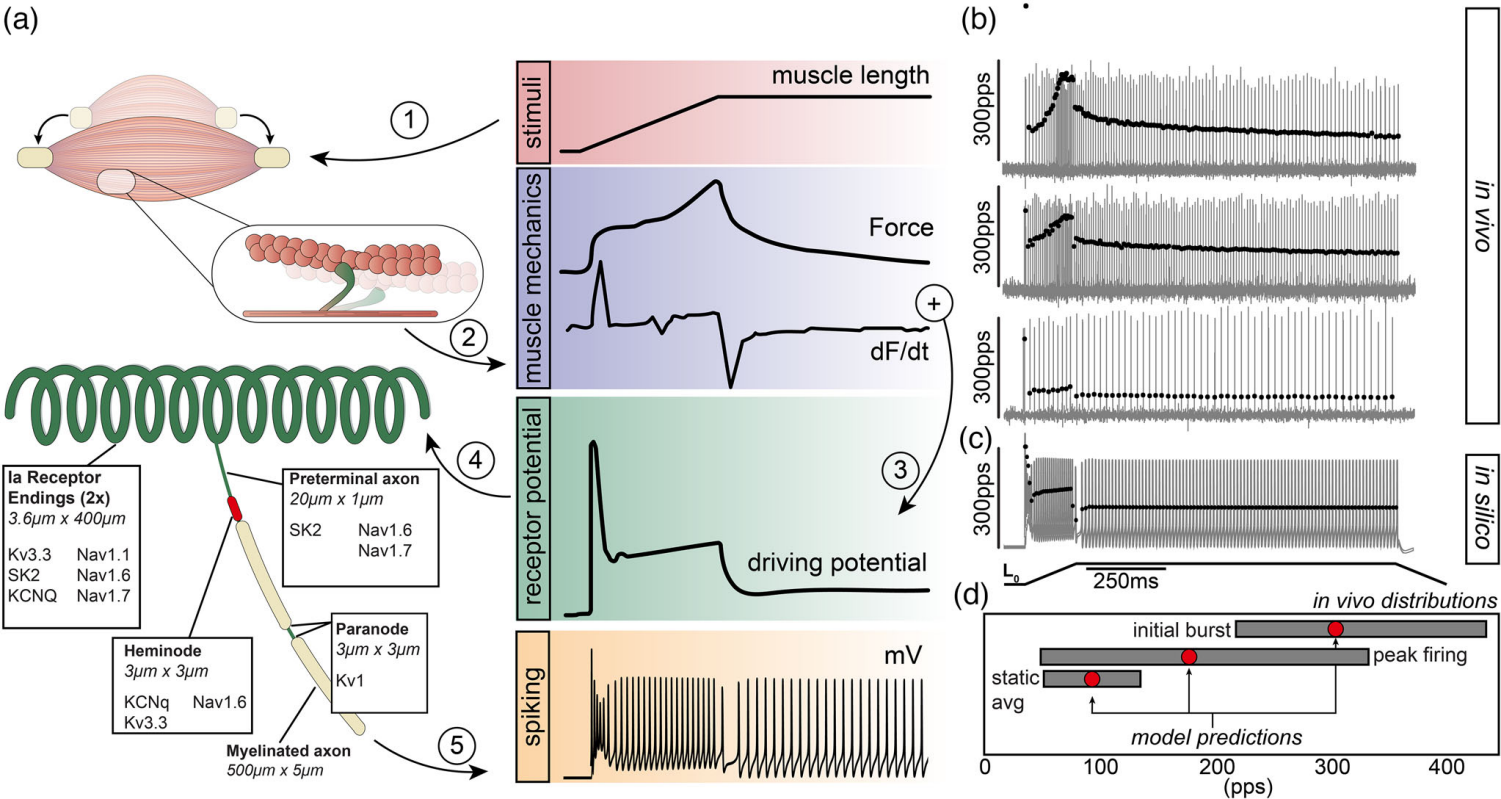
Biophysical modelling of spindle Ia afferents. (a) Overview of the methodology used to model Ia muscle spindle firing. Physiological stimuli (ramp–hold–release), recorded during in vivo experiments, were used as inputs (1) to a biophysical model of muscle cross‐bridges to simulate intrafusal fibre dynamics (2). Two components of muscle fibre dynamics were then combined to estimate the muscle spindle Ia receptor potential (conductance) as described in Blum et al. (2020) (3). The receptor potential was the input into the multicompartment biophysical model of the neuronal architecture and ion channel expression and distribution (4) that simulates neural dynamics, for example, spiking (5). (b, c) Three representative cases of spiking activity and corresponding instantaneous firing rates (vertical grey lines and black circles) for Ia neurons recorded in vivo (b) and corresponding model predictions (c). Note the wide biological variability of Ia neurons’ firing profiles and the fact that the model output is well situated within that range. (d) Grey rectangles document the minimum and maximal values of three key encoding parameters recorded in vivo (Housley et al., 2020): average static firing rate (static avg.), peak dynamic firing rate (peak firing) and initial burst. Red circles document model output for same key parameters.

We developed a bespoke multi‐compartment biophysical neuron model implemented in NEURON 8.2 to simulate the behaviour of the prototypical rat muscle spindle Ia afferent. Microscopy and electrophysiological experiments provide prior evidence on the detailed morphology and approximate distribution of established currents with which we modelled sub‐compartments of the muscle spindle Ia afferent (Bewick & Banks, 2021). The distribution of conductances and their parameters are described in Figure 1a and Table 1. Briefly, the model contains critical components that control the receptor potential amplification and pattern of discharge including: a Kv1 channel model using HH‐type activation/inactivation; a voltage‐gated Kv3 channel with high threshold and fast activation/deactivation kinetics; a slowly inactivating, calcium‐dependent potassium channel responsible for medium‐duration (SK2); a KCNQ (KM‐current), 10‐state Na_v_1.1 channels with two slow inactivation states; a 10‐state Na_v_1.6 kinetic gating model with two slow inactivated states, one entered from the open state and one from a fast‐inactivated state; and a six‐state Na_v_1.7 HMM channel. The model, underlying ion‐channel models, and associated input/output files will be uploaded to ModelDB (https://senselab.med.yale.edu/ModelDB/) upon publication. Each neuron model configuration was fitted to a prototypical rat muscle spindle Ia response. The prototypical Ia response included dynamic‐phase and static‐phase firing rates (*n* = 11).

**TABLE 1:** Voltage‐gated channel distribution and base conductance across four anatomical regions.

Ion channel (conductance)	Ia receptor ending	Preterminal axon	Heminode	Paranode
Na_v_1.1 (pS/μm^2^)	250	0	0	0
Na_v_1.6 (pS/μm^2^)	25	250	2500	0
Na_v_1.7 (pS/μm^2^)	250	500	0	0
Kv1 (S/cm^2^)	0	0	0	0.01
Kv3.3 (S/cm^2^)	0.012	0	0.012	0
SK2 (S/cm^2^)	0.0025	0.0025	0	0
KCNq (mho/cm)	0.0005	0	0.0005	0

#### 2.2.4 Model perturbations

We systematically varied the maximal conductance of pairs of ion channels to reveal regions in parameter space associated with prototypical Ia firing responses. For a given combination of conductance values, we sampled a wide distribution to best validate model performance on the distribution of experimental recordings. We also tested the consequences of systematically altering neuroanatomical architecture by varying model configurations bounded by the range of recorded dimensions from past studies.

## 3 RESULTS

### 3.1 Biophysical modelling of wild‐type muscle spindle Ia afferents

We set out to simulate Ia spike encoding by constructing a computational model with three primary modules: (1) a biophysical model of intrafusal muscle fibre dynamics that respond to physiologically relevant stimuli, (2) a generator function that sums kinetic elements of the intrafusal fibre dynamics to estimate a receptor potential, and (3) a biophysical representation of neuronal architecture and ion channel type and distribution (Figure 1a). The neuronal dynamics model is the focus of this work and comprises multiple compartments representing sequential information flow from mechanotransduction to spike generation: two receptor terminals (annulospiral) converging on a single preterminal axon which is directly connected to five nodes of Ranvier (first one defined as ‘heminode’) serially arranged and separated by myelinated axon segments (Figure 1a). Paranode segments are immediately adjacent to nodes (see Methods). Base model parameters of neuronal architecture are derived from measures of muscle spindle Ia terminals in rat. The expression of ion channels in the base model is derived from diverse pharmacological, immunohistochemical and genetic studies across both rat and mouse (Bewick & Banks, 2015; Bewick & Banks, 2021; Bewick et al., 2005; Carrasco et al., 2017; de Nooij et al., 2015; Housley et al., 2020; Simon et al., 2010; Woo et al., 2015).

The base model reproduced prototypical Ia firing dynamics in response to a physiological ramp–hold–release stimulus. Firing properties that were well‐fitted by the base model included a high‐frequency response at stretch onset (initial burst), accelerating firing rates with increasing stretch amplitude (dynamic firing), and an adapted firing rate during the static phase of stimuli (Figure 1b). Given the diversity of potential firing profiles among Ia afferents, we did not fit base model performance to a single in vivo recording (Figure 1b). Instead, we demonstrate that the base model generates a firing pattern situated within the range of Ia responses observed in vivo (Figure 1c; three representing a typical range are shown) as well as values for key firing parameters derived from the extensive database of in vivo recordings from our lab (Figure 1d).

### 3.2 Computational simulations predict that muscle spindle architecture can account for differences in firing characteristics between Ia afferents

Taking advantage of the reconfigurable computational model, we tested the hypothesis that neuronal architecture influences Ia firing responses. We compared predicted firing rates during ramp–hold–release stimuli for ranges of receptor terminal and preterminal axon lengths while all other parameters were held constant. The range of terminal lengths selected for evaluation are derived from detailed measures (unpublished rat data) that encapsulate the range of expected dimensions across different species, for example, mouse, rat, cat, human. Comprehensive experimental data on the range of preterminal axon dimensions does not exist, so we simulated the effects of altering preterminal length across a 12.5‐fold range above and 4‐fold range below base model dimensions to include the majority of neuronal architectures expected biologically.

**FIGURE 2:**
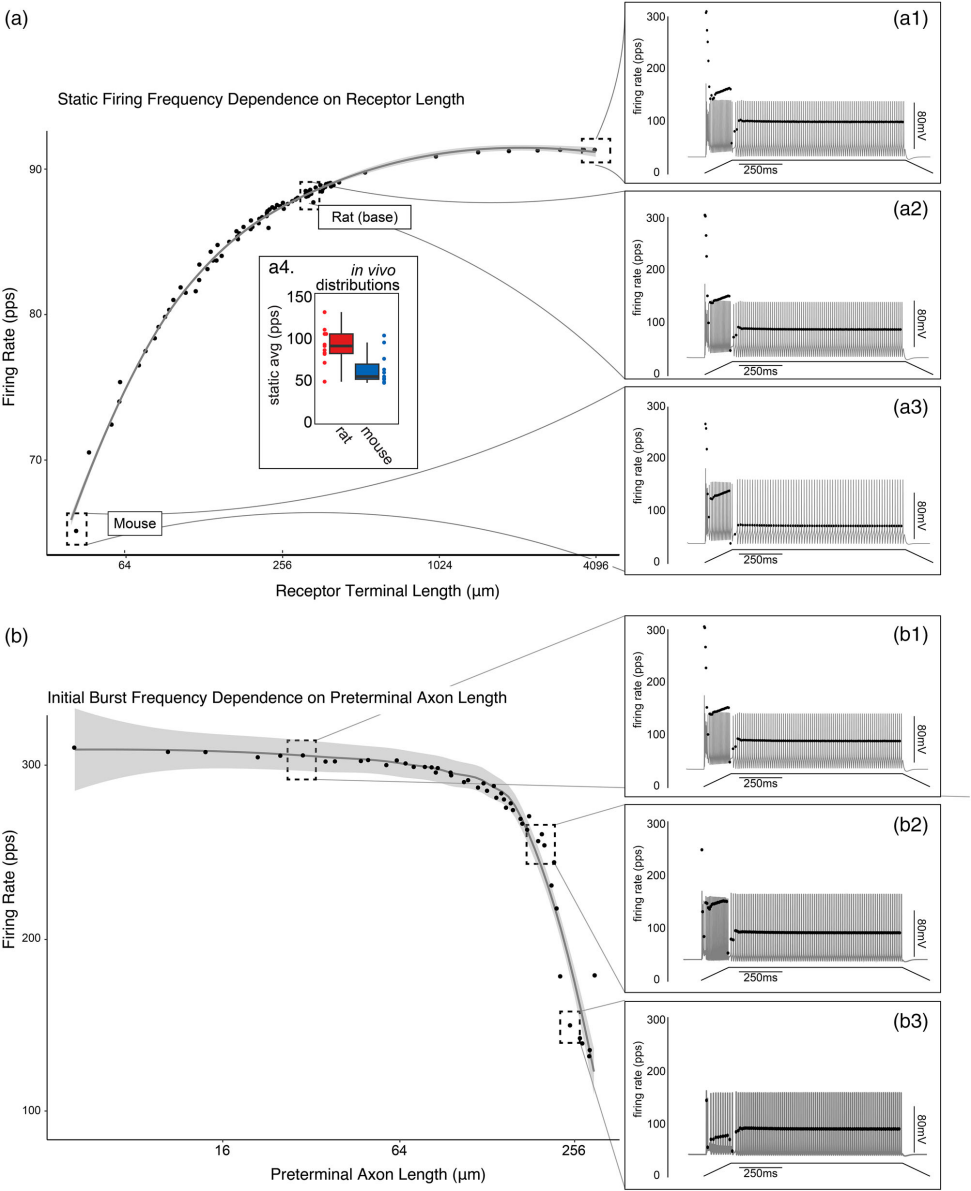
Muscle spindle architecture can account for differences in Ia firing characteristics. (a) Relationship between model Ia terminal length and average static firing rate plotted on a log_2_ scale. Three dashed boxes expanded to show the full modelled firing profile across the range of model configurations (a1–3). Inset presents average static firing rate recorded in vivo for both rat (red) and mouse (blue). (b) Relationship between model preterminal length and initial burst frequency plotted on a log_2_ scale. Three dotted boxes expanded to show the full modelled firing profile across the range of model configurations (b1–3).

We found that increasing terminal length (from base model) by 10‐fold had a nominal effect on key firing parameters including initial bursts (0.764%), peak dynamic firing rate (1.54%) and static firing rate (2.28%) (Figure 2a). Conversely, decreasing terminal length by the same magnitude drastically reduced the same firing parameters: initial burst (12.6%), peak dynamic firing rate (14.1%) and static firing rate (27.7%) (Figure 2a). While likely an incomplete story of scaling among species, the validity of firing rates predicted by our biophysical model when we scale terminal morphology ranges between those observed for both rat and mouse is supported by differential static firing rates observed in both species in vivo (Figure 2a4). Overall, we found that terminal length affected all components Ia encoding but influenced static firing to a greater extent.

In contrast to terminal length, systematically manipulating preterminal axon length had more restricted effects on Ia firing (Figure 2b). Preterminal axon morphology exclusively reduced initial burst frequencies (17.1%) out to 10‐fold longer lengths as compared to 1.87% and 2.01% reductions in dynamic and static firing parameters. All firing parameters were invariant to shorter preterminal axon lengths (ranging from 0.178% to 1.55% change). Unexpectedly, increasing preterminal axon length beyond the 10‐fold threshold (as little as 10–20 μm) revealed a critical threshold where peak dynamic firing and initial burst frequencies drastically collapsed (57.6% and 52.5%, respectively; Figure 2b3). Simulations also predict that linearly increasing preterminal axon length results in phase shifts in dynamic firing patterns transitioning to bi‐stable and then ultimately to irregular/stochastic firing patterns.

Collectively, this model predicts that neuronal architecture can play a role in determining muscle spindle encoding. Terminal morphology acts as a general amplifier, while the preterminal axon morphology selectively filtering initial burst and dynamic firing characteristics.

### 3.3 Computational simulations predict that muscle spindle encoding is robust across a wide range of ion channel expression values

**FIGURE 3:**
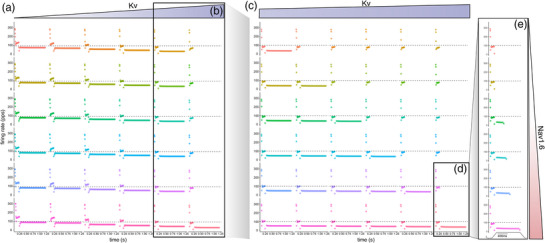
Muscle spindle Ia firing across a wide range of ion channel expression values. (a) Systematic manipulation of voltage‐gated KCNQ (Kv) and slowly inactivating Na^+^ Nav1.6 channels across a wide 2D conductance range (Nav1.6: 0–50 pS/um^2^ and Kv: 0.0005–0.005 S/cm^2^) in Ia terminals inclusive of the base model. Grids showcase modelled instantaneous firing rates for each model configuration of a specific Kv (horizontal: blue) to Nav1.6 (vertical: red) ratio. Sequential insets document narrowing conductance spaces. (b, c) Narrow range of Kv expression that results in loss of static firing (b), which is the focus of expanded simulations in (c). (d, e) At one phase transition, the final detailed simulation (d) revealed that the complete spectrum of static firing is expressed across a narrow ∼2 pS/μm2 Nav1.6 range (e).

Next, we tested the functional contributions of the asymmetric distribution of VGCs on Ia firing patterns. Given the fact that 2^13^ binary permutations (i.e., channel is present or absent in one of the model segments) are possible (not to mention the continuous possible conductance spaces for each permutation), we constrained our investigation by systematically manipulating two conductance in receptor terminals: voltage‐gated KCNQ (Kv) and Na_v_1.6 channels. An initial sweep across a wide 2D conductance range inclusive of the base model (Na_v_1.6: 0–50 pS/μm^2^ and Kv: 0.0005–0.005 S/cm^2^) revealed a 49.4% reduction in peak dynamic firing rates under the lowest Na_v_1.6/Kv ratios studied. While static firing rate scaled positively and expectedly as Na_v_1.6 increased and Kv decreased, static firing rate depended to a larger extent on Kv expression (Figure 3a). At the highest Kv values studied, static firing ceased entirely and was only restored, albeit at the lowest firing rates, that is, 36.6 pps (a 59.0% drop) by the high Na_v_1.6 conductance. Surprisingly, initial bursts remained only mildly impacted by large deviations in the balance of Na_v_1.6/Kv with 7.69% reductions at the highest Kv and lowest Na_v_1.6 expression levels.

To further investigate the dependence of static firing rate on the balance of Na_v_1.6/Kv, we performed sequential, 2D sweeps through narrowing conductance spaces to find the range where static firing transitioned from competent to incompetent to sustain firing. Figure 3b highlights the narrow static firing phase transition across the diagonal that depends on a <5% Kv change as Na_v_1.6 conductance increases. A final detailed simulation centred at one of these phase transitions (Figure 3c) revealed that the complete spectrum of static firing is expressed across a narrow ∼2 pS/μm^2^ Na_v_1.6 range (Figure 3d). Collectively, these results lead to two predictions. First, muscle spindle firing is robust across a wide range of conductance, scaling as Na_v_1.6 and Kv conductance change, leaving the general profile largely intact. Second, static firing is more sensitive to changes of conductance in receptor terminals, expressing a steep phase transition over a narrow band of Na_v_1.6.

### 3.4 Computational simulations predict that muscle spindle receptor is competent to generate and support AP initiation

**FIGURE 4:**
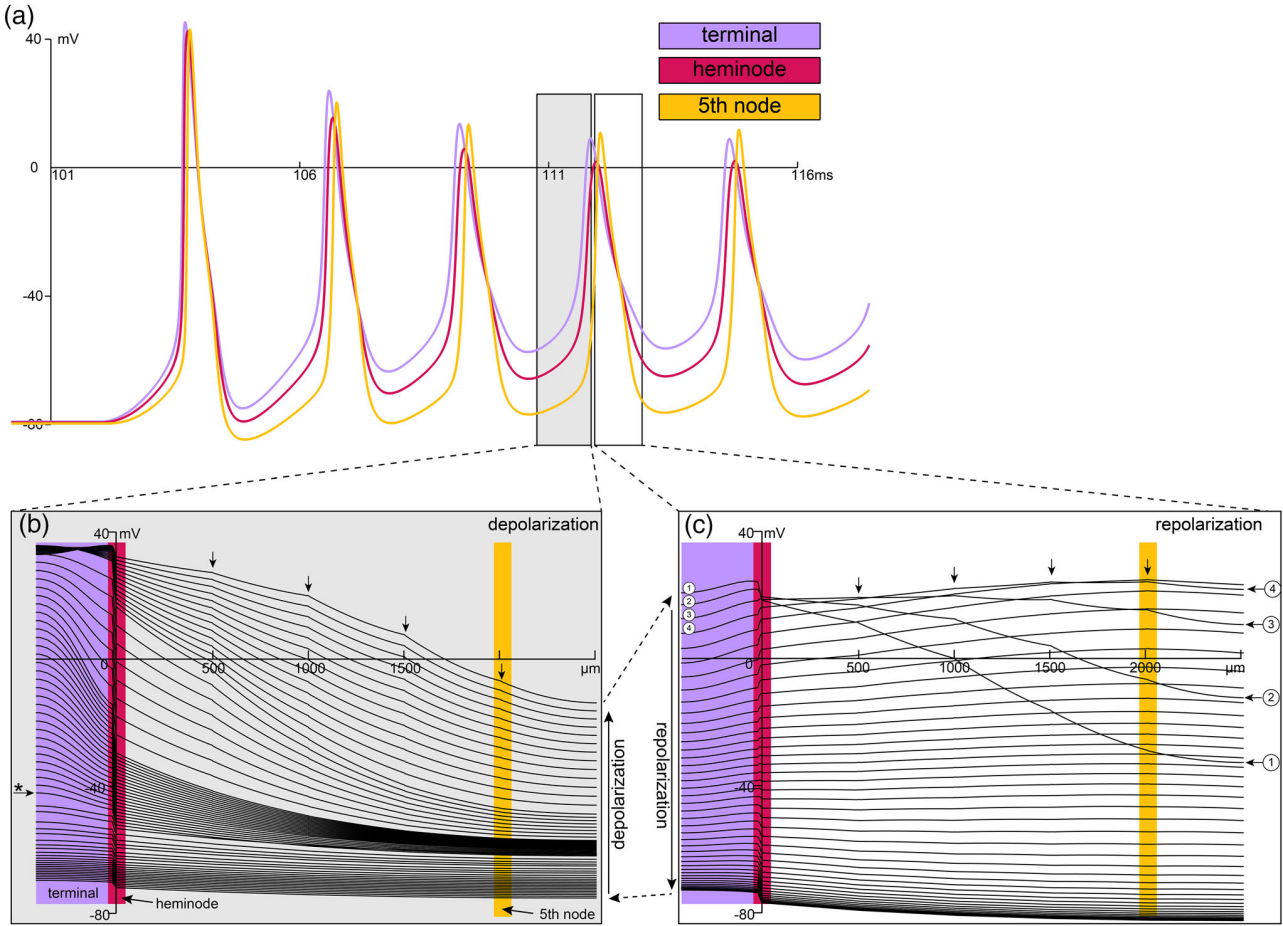
Spike generation in Ia muscle spindles. (a) Voltage versus time outputs for the first five action potentials (AP) as the model responds to physiological ramp–hold–release stimuli (identical to all other simulations: see Figure 1). Three traces are colour‐coded to indicate key spatial domains in the generative model: Ia terminals, heminode, and fifth node of Ranvier. (b, c) plots of the voltage over the entire length of the model for the fourth AP. Grey (b) and white (c) insets show the depolarization and repolarization by space of their corresponding time courses shown in (a). Each line indicates a single snapshot in time as the model determines the voltage as it solves the joint system of differential equations governing the constellation of channel kinetics. (b) starting from the bottom left and moving up, the wave of depolarization reaches threshold, that is, AP is initiated in the Ia terminals as indicated by the inflected voltage trajectory (* horizontal arrow) and transition to steep voltage versus space relationship. The AP reaches maximum depolarization by the top trace. (c) rapid reversal of Ia terminal AP as shown in the first four traces (numbered). AP propagation initially to the heminode followed by a rapid depolarization then repolarization through the remaining nodes of Ranvier (four vertical arrows).

Given the constellation of ion channels expressed in muscle spindle receptor terminals, including those that support AP generation and repetitive firing in other systems, we performed simulations to investigate the dynamics of spike generation in our biophysical model. Figure 4a plots voltage versus time colour‐coded across three key spatial domains in the generative model: receptor terminal, heminode and fifth node of Ranvier. Simulations show the first five APs as the model responds to the dynamic portion of identical ramp‐hold‐release stimuli. It predicts that the first AP is initiated in the receptor terminal, followed closely by the heminode and then fifth node AP.

Expanding on this discovery, we next plotted the voltage over the continuous model space for the fourth AP (Figure 4b,c). Grey (Figure 4b) and white (Figure 4c) insets show the depolarization and repolarization trajectories where each line indicates the instantaneous voltage recorded across the entire space for a specific time step and the colour gradient indicates location along the model. Starting from the bottom left in Figure 4b and moving up, the wave of depolarization (i.e., AP) initiates in receptor terminals as indicated by the steep inflection of the voltage trajectory (Figure 4b: horizontal arrow) before reversing rapidly as shown in the first four traces in Figure 4c (numbered). This transition shows the AP propagation initially to the heminode followed by a rapid depolarization then repolarization event through the remaining nodes of Ranvier (four vertical arrows in Figure 4b,c). The results of this biophysical simulation lead us to predict that the muscle spindle receptor terminal is competent to generate APs and may be capable of self‐sustained repetitive firing. While it remains to be seen if APs occur in muscle spindle terminals in vivo, these computational results when combined with prior knowledge of expressed VGCs elevate the potential.

## 4 DISCUSSION

Our objective was to begin constructing the first integrative biophysical model of muscle spindle firing. We leveraged current knowledge of muscle spindle neuroanatomy and in vivo electrophysiology to develop and validate a biophysical model that reproduces key in vivo muscle spindle encoding characteristics. To our knowledge, this is the first computational model of mammalian muscle spindle that integrates the asymmetric distribution of known VGCs with neuronal architecture to generate realistic firing profiles, both of which seem likely to be of great biophysical importance. Results predict that particular features of neuronal architecture regulate specific characteristics of Ia encoding. Computational simulations also predict that the asymmetric distribution and ratios of VGCs is a complementary and, in some instances, orthogonal means to regulate Ia encoding. Specifically, static firing rates vary with Ia terminal length across a 10‐fold range that covers lengths observed in multiple species. Beyond that, static firing rates become invariant to longer terminal lengths. Preterminal axon length specifically impacted dynamic firing. Static firing depends on a tightly controlled ratio of Kv/Na_v_, with high ratios resulting in complete loss of static encoding. These results generate testable hypotheses and highlight the integral role of peripheral neuronal structure and ion channel expression in somatosensory signalling.

### 4.1 Structural determinants of muscle spindle firing

Mechanosensory receptors display a rich array of end organ morphologies that correlate with distinct physiological functions. However, understanding of the ways in which specific features of Ia neuronal architecture influence firing patterns is incomplete (Chalfie, 2009). Prior computational studies in tactile sensory neurons (slowly adapting type I afferents) indicated that both the number of transduction units and their arrangement can regulate slowly adapting type I afferents afferent firing properties (Lesniak et al., 2014). Our computational results mirror this discovery by implicating muscle spindle neuronal architecture as a key regulator of encoding properties. We find that Ia terminal morphology (length) acts as a non‐specific amplifier of Ia firing properties (Figure 2a). In this way, terminal morphology is another means to tune Ia encoding to complement or compensate differential VGC expression. It has been assumed that mechanical properties are fully responsible for initial burst behaviours (Proske et al., 1993) expressed by type Ia muscle spindles but present findings from this model demonstrate that preterminal axon morphology has the potential to selectively filter high frequency firing rates at the onset of stimuli (Figure 2b).

### 4.2 Utility of integrative biophysical model of muscle spindles

While intrafusal fibre mechanics, neuronal architecture and neural dynamics are inextricably linked in vivo, prior muscle spindle models have focused on isolated components or utilized entirely phenomenological models (Blum et al., 2020; Hasan, 1983; Lin & Crago, 2002; Mileusnic et al., 2006; Schaafsma et al., 1991). Prior models may have been capable of fitting prototypical Ia firing characteristics, but adopting reduced computational or purely empirical models limits the suite of biologically relevant hypotheses that can be generated. In particular, prior to the present study, it was not possible to test the functional role specific VGCs played in muscle spindle encoding. Genetic perturbations offer a window into their putative role but compensatory mechanisms complicate interpretation (El‐Brolosy & Stainier, 2017). Thus, biophysical modelling has the potential to give much greater insight and resolution to the role of specific components of the mechanosensory unit. The utility of biophysical modelling is further elevated when considering the need to study mechanisms not in isolation but as they interact. These situations occur frequently in various disease states (Housley et al., 2020) or when attempting to determine somatosensory encoding across biological scales in comparative studies. Thus, computational modelling is a powerful ally of transgenic approaches whereby specific targets can be prioritized making more efficient use of the time‐ and resource‐intensive experimental techniques. Another limitation of prior approaches has been to restrict a model's adaptability to be rapidly updated as new discoveries are made. Instead, by incorporating modular biophysical components, the present model was designed to accommodate the anticipated discovery of new VGCs as well as other key mechanisms in spindle input/output operations.

### 4.3 Diversity of voltage‐gate ion channels

It seems likely that most VGCs will play important roles in shaping and modulating muscle spindle responses. However, the current study by necessity is a first step and only examined a restricted number of VGCs present. Given the sheer number and still expanding list of VGCs expressed in muscle spindles, a persistent question emerges: what is the functional role that each VGC plays? This would be difficult and very time‐costly to interrogate purely using genetic models and, with their intrinsic difficulties of compensation, may not provide definitive answers. The reconfigurability of our computational model allows us to take the first steps in answering this question.

One such step is that by removing (‘knocking‐out’) specific sodium channel isoforms known to be expressed in receptor terminals, we find that both Na_v_1.6 and Na_v_1.7 are equally competent to support canonical Ia firing. While the reason for such biological redundancy is not known, we propose two potential explanations. First, as muscle spindles are the source of mechanosensory information generation, it is reasonable to suspect that having a high biophysical safety factor, that is, not a single point of failure, is needed to ensure a robust response is mounted to mechanical perturbation. Second, it is increasingly appreciated that VGCs influence cell behaviour in ways other than to support cellular excitability. Non‐conductive functions (Kaczmarek, 2006), for example, include various inter‐ and intracellular signalling pathways such as cytoskeletal remodelling, cell motility and adhesion, and ability to influence expression of specific genes (Cai et al., 2005, 2009; Dolmetsch et al., 2001; Kaczmarek, 2006; Mochida et al., 1998; Runnels et al., 2001) or may even be tied to an autogenic or extrinsic feedback mechanism. It is possible that one or other of these channels has additional roles yet to be uncovered. As discovered, these specialized functions will be incorporated into the model.

### 4.4 Limitations

Despite the advancements made by, and utility of, the present modelling approach, a number of limitations need mentioning.

First, the neuronal architecture was largely based on muscle spindles reconstructed from rat triceps surae muscles. Detailed reconstructions are needed from different muscles, for example, non‐antigravity muscle, and from different experimental species. A recent survey of mouse muscle spindle provided boundaries for morphological simulations (Lian et al., 2022) but was restricted to receptor terminal length and number. The authors did not report receptor terminal diameter or any other neuroanatomical feature, for example, preterminal morphology.

Second, while the present study is the first to incorporate a comprehensive constellation of asymmetrically distributed VGCs, we treat the density of ion channels in a given segment in the multicompartment model as uniform. This assumption seems likely to be wrong yet is based on the best available evidence that has merely identified the presence of a given VGC in a segment. Additional studies are underway in our lab to clarify the detailed subcellular distribution and to quantify the differential ion channel densities across the neuronal architecture.

Third, for computational simplicity, we restricted the neuronal architecture to include two Ia terminals converging on a single heminode via a shared preterminal axon. The biological diversity of muscle spindle morphologies is well documented (Banks, 1994; Banks et al., 1982, 1997; Hulliger, 1984). Given the role that different configurations of mechanotransduction sites and spike initiating zones play in tactile sensory encoding, future iterations of this model need to incorporate different ratios and distances of Ia terminals and heminodes. In addition, future model iterations will need to explicitly incorporate competitive pacemaker interaction between encoding sites (Banks et al., 1997).

Finally, the integrative biophysical model presented here is merely a starting place. As data continue to emerge on the constellation of VGCs as well as other key mechanisms in spindle input/output operations, the model must be updated. In particular, work has already begun on incorporating autogenic feedback mechanisms and non‐uniform VGC densities, which were explicitly left out of the present study, in favour of establishing a simple baseline model on which to build such complexity.

## AUTHOR CONTRIBUTIONS

Computational modeling: Stephen N. Housley, Randal K. Powers, Kyle Blum. Afferent Recordings: Paul Nardelli. Morphometeric analysis of voltage gated ion channels: Sebinne Lee, Stephen N. Housley. Analysis and interpretation of data, Stephen N. Housley, Randal K. Powers, Paul Nardelli, Sebinne Lee, Guy S. Bewick, Robert W. Banks, Timothy C. Cope. Drafting or revising the article: Stephen N. Housley, Timothy C. Cope, Randal K. Powers, Guy S. Bewick, Robert W. Banks. All authors have read and approved the final version of this manuscript and agree to be accountable for all aspects of the work in ensuring that questions related to the accuracy or integrity of any part of the work are appropriately investigated and resolved. All persons designated as authors qualify for authorship, and all those who qualify for authorship are listed.

## CONFLICT OF INTEREST

The authors declare that they have no known competing financial interests or personal relationships that could have appeared to influence the work reported in this paper.
